# Second-derivative UV spectral analysis of aqueous humor for eye disease diagnosis and assessing the effects of food additives on ocular health

**DOI:** 10.1038/s41598-025-02190-w

**Published:** 2025-05-19

**Authors:** Sherif S. Mahmoud, Shimaa M. Elshebley, Eman M. Aly, Sahar M. Awad, Gehan M. Kamal

**Affiliations:** 1 Biophysics and Laser Science Unit, Research Institute of Ophthalmology, Al Ahram Street, Giza, Egypt; 2 Physics Department, Faculty of Science, Al-Azhar University (Girls branch), Cairo, Egypt

**Keywords:** Eye, Aqueous humor, UV spectroscopy, Second derivative, Chemometric analysis, Biophysics, Biomarkers, Diseases, Health occupations

## Abstract

Ultraviolet absorption (UV) spectroscopy is an invaluable method for analyzing compounds in ocular fluids, identifying chemical structures, and understanding molecular interactions. The widespread use of additives in food production is well documented; these additives color, flavor, preserve, and enhance the texture or nutrition of food, ensuring a broader range of products and reducing costs. This study employed UV spectroscopy to examine changes in aqueous humor composition after daily intake of permitted amounts of food additives over 45–90 days. The rats were administered colorant dyes (carmoisine or tartrazine), a sodium benzoate preservative, or a combination of all three through oral gavage. Aqueous humor samples were collected post sedation from the anterior chamber via a 30-gauge needle without additional processing. The spectra of these samples were analyzed via secondary derivative calculations and chemometric analysis (principal component and hierarchical analyses) with OriginPro 2015 software. The common outcome observed was ocular toxicity, resulting from decreased antioxidant defense mechanisms, leading to ocular side effects on the cornea and lens. These changes in aqueous humor composition are indirectly linked to food additives rather than the mechanisms of aqueous humor formation.

**Supplementary Information:**

The online version contains supplementary material available at 10.1038/s41598-025-02190-w.

## Introduction

The composition of aqueous humor varies significantly across different stages of life and under various eye conditions. Studies have shown that changes in aqueous humor dynamics can impact intraocular pressure (IOP), with significant differences in lipid and amino acid metabolism^1,2^. Additionally, the chemical composition of aqueous humor plays a crucial role in maintaining corneal health and clarity, with an abnormal composition linked to corneal dystrophies and endothelial dysfunction^3^. Furthermore, variations in element concentrations, such as those of sodium, potassium, nitrogen, and sulfur, have been observed in the aqueous humor of individuals with different levels of intraocular pressure, highlighting the dynamic nature of aqueous humor composition^4^. Moreover, heightened levels of electrolytes in the aqueous humor of individuals with cataracts might contribute to the onset of this condition^5^, and changes in lens composition could conceivably be the result of changes in aqueous humor composition^6^.

Examining changes in aqueous humor composition has emerged as a promising diagnostic tool for a range of eye ailments. Studies have indicated notable shifts in metabolite profiles in patients with macular edema (ME) of diverse origins, aiding in early detection and personalized treatment approaches^5^. Furthermore, investigations in individuals with type 2 diabetes have pinpointed specific metabolic and proteomic alterations in the aqueous humor, suggesting its potential as a gauge of disease severity and a tool for assessing risks and future therapies^2,7^. Additionally, research into normal-tension glaucoma (NTG) has revealed differentially expressed proteins in the aqueous humor that correspond to functional and structural parameters, suggesting potential molecular markers for advanced NTG diagnosis^8^. Collectively, these findings underscore the significant promise of analyzing aqueous humor composition in the diagnosis and management of various eye conditions.

On the one hand, using food additives lies in their ability to enhance food quality, safety, and shelf-life, playing crucial roles in maintaining the organoleptic properties of food^9,10^. However, concerns exist regarding the potential health impacts of additives, emphasizing the need for continuous monitoring and risk assessments to safeguard public health^11^. The consumption of food additives can significantly impact ocular health. Synthetic chemicals utilized as food additives have been linked to a range of health issues, particularly concerning the eyes. These include dry eye disorders, myopia progression, cataracts, glaucoma, diabetic retinopathy, and age-related macular degeneration. Furthermore, synthetic colorant additives and preservatives can disrupt hormone balance, impede growth and development, and contribute to obesity, all of which can detrimentally affect eye health. Additionally, some food preservatives have been found to alter the expression of inflammatory molecules in tissues, potentially influencing ocular health^12–15^.

This study provides a practical approach that can be readily implemented in clinical settings to examine the composition of aqueous humor without any sample processing. This involves recording the UV spectra and analyzing their second derivatives. The study investigated the effects of consuming various food additives, including colorants (carmoisine and tartrazine), preservatives (sodium benzoate), and their combination, on the composition of aqueous humor after 45 and 90 days. Chemometric analysis was also employed for further analysis.

## Materials and methods

### Materials

A total of 125 adult albino Wister rats (65 males, 60 females) weighing between 150 and 200 g were used for this research. These animals were randomly selected from the animal housing facility at the Research Institute of Ophthalmology (Giza, Egypt). They were maintained in a controlled environment with a 10-hour light and 14-hour dark cycle at a temperature of 25 °C and 40% humidity, ensuring adequate ventilation, and were provided with a standard diet throughout the experiment. The handling of the animals followed the guidelines set forth by the Association for Research in Vision and Ophthalmology (ARVO) regarding the use of animals in ophthalmic research. Additionally, the research protocol received approval from the local ethical committee of Research Institute of Ophthalmology (RIO-Committee), the study is reported in accordance with ARRIVE guidelines.

Food additives were sourced in powder form from the Cairo Food Company (Luna Company, Giza, Egypt). Tartrazine, a yellow coloring agent (C.I. No. 19140, also recognized as E102 or FD&C Yellow 5), exhibited a purity of 96.8%. Carmoisine, a red maroon colorant (C.I. No. 14720, also known as azorubine, E122, acid red), had a purity of 95.8%. Additionally, sodium benzoate, a white preservative salt (known as E211), was acquired with a purity of 95%. The applied dosage of food additives adhered to the acceptable daily intake (ADI) guidelines established by the World Health Organization (WHO) and the European Food Safety Authority^16–19^. The daily ADI dosage of tartrazine is 7.5 mg/kg body weight, carmoisine is 4 mg/kg body weight, and sodium benzoate is 5 mg/kg body weight.

### Administration of food additives

The rats were randomly allocated into ten groups, each comprising either 10 rats (for the 45-day study period) or 15 rats (for the 90-day study period). Food additives were administered to the rats via stomach tube, which were dissolved in bi-distilled water. The specified doses of the three food additives were administered daily for either 45 days (in three groups) or 90 days (in three groups). Additionally, two groups, termed the ‘Mixture groups’, received an oral mixture of all three food additives and were studied after both 45 and 90 days. The remaining two groups served as controls for the same durations and received 1 ml of bi-distilled water via a stomach tube, mirroring the method used for the treated groups.

Note that these time windows were chosen based on the metabolic cycle and cumulative effects of toxicity in rodent models. The 45-day period corresponds to approximately six weeks, allowing for the assessment of sub-chronic effects and early physiological adaptations to the tested food additives. The 90-day period aligns with Organization for Economic Co-operation and Development (OECD guidelines, https://www.oecd.org/chemicalsafety/testing/OECDTG408) for repeated-dose toxicity studies and provides insights into long-term cumulative toxicity effects. Additionally, this duration enables evaluation of potential organ-specific toxicity and metabolic disturbances that may arise over extended exposure. The sample size was calculated based on the minimum number of animals to meet the desired statistical constraints and found to be 10–15 rats are needed to have a confidence level of 95% within ± 5% margin of error. Experimental grouping was randomized and data collection was blind.

### Aqueous humor sampling

At the specified periods and after intramuscularly induced anesthesia with ketamine (87 mg/kg) and xylazine (13 mg/kg), a syringe equipped with a 30 g needle was inserted into the anterior chamber of each rat. Approximately 3–5 µl of aqueous humor (AH) was withdrawn from both eyes and immediately, without any further manipulations, subjected to UV spectroscopy. Note that, rats were killed by intravenous administration of sodium pentabarbitone (200 mg Kg^− 1^).

### Recording UV absorption spectra

A UV‒vis spectrophotometer (Evolution 600, Thermo Fisher, USA) and a quartz cuvette with a 1 mm light path and 10 mm width were used; the absorption spectra were acquired at room temperature (25 °C) with five-multiple scans between 190 and 400 nm (with a fixed 5 nm bandwidth) and subsequently averaged. Measurements were recorded against distilled water as the blank. The second derivative of the UV spectra was calculated using OriginPro 2015 software (OriginLab Corporation, Northampton, MA, USA). Careful analysis of these derivatives was performed using the curve fitting tools of OriginPro 2015 software, where the peak setting parameters were set to fit the negative peaks. Accordingly, the area under each peak was calculated (baseline correction method) and used in this analysis.

### Statistical analysis

The data are presented as the means ± standard deviations, with the significance level set to *p* < 0.05 using one-way ANOVA test. Levene’s test was used to assess the homogeneity of variances while, multiple means comparisons between groups were conducted using Bonferroni test.

Multivariate analysis was performed via the statistical tools of OriginPro 2015 software, including principal component and hierarchical cluster analyses.

## Results

UV spectroscopy is a simple, cost-efficient and provides rapid detection of molecular components while, its second derivative spectra can discriminate the overlapping of absorption peaks, thus improving peak resolution and reducing baseline noise, enhancing the differentiation of closely related compounds. These characteristics enabling its potential application for rapid-medical monitoring of diseases especially in situations where time-interference is an important factor, and providing a potential tool for non-invasive diagnosis of eye diseases Altogether, making its use is preferred when compared with more precise measurements as mass spectroscopy; in which an expensive instrument, special sample preparation steps, and highly-skilled operator are required.

### Typical UV spectra of aqueous humor (AH)

The absorption spectrum of aqueous humor in the ultraviolet‒visible (UV‒Vis) spectroscopy range can vary depending on factors such as age, health status, and environmental exposure. However, there are certain absorption peaks that can be associated with proteins, tryptophan, tyrosine, ascorbic acid, and uric acid.

Proteins and amino acids generally exhibit characteristic electronic absorption patterns in aqueous environments, displaying broad features within the UV region of the electromagnetic spectrum (190–370 nm). The distinct absorption peaks, typically observed between 255 and 280 nm, are attributed to chromophores found in the side chains of aromatic amino acids such as tryptophan (Trp, approximately 280 nm), tyrosine (Tyr, approximately 275 nm), and phenylalanine (Phe, approximately 257 nm). Furthermore, the peptide bonds within proteins display a strong absorption peak around 190 nm, along with a weaker absorption band between 210 and 220 nm. The absorption peaks between 320 nm and 370 nm originate from the conjugated groups C=C and C =O, respectively^20,21^.

**Fig. 1:**
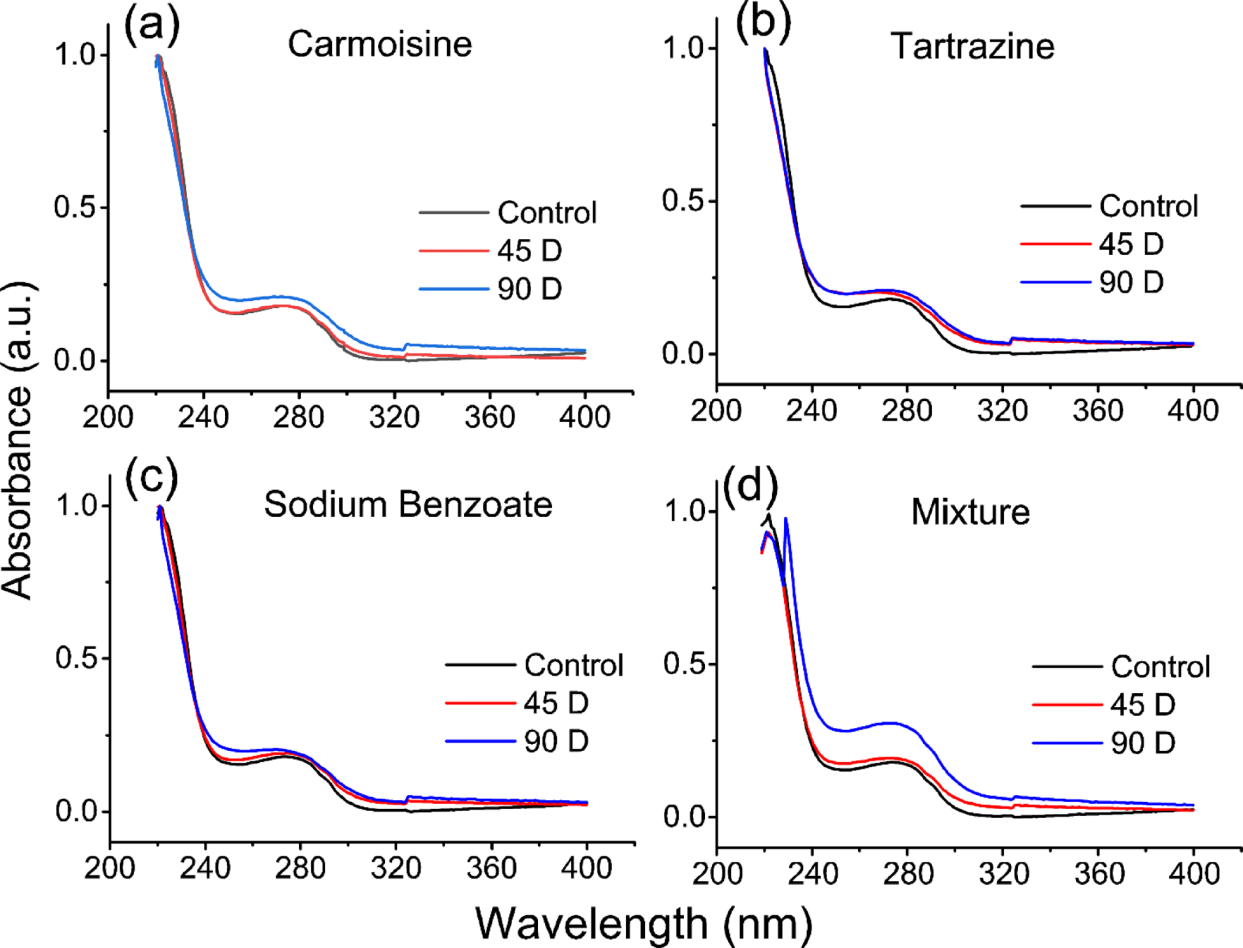
Typical ultraviolet spectra of aqueous humor fluids collected from the control groups and (**a**) carmoisine, (**b**) tartrazine, (**c**) sodium benzoate, and (**d**) their mixture after daily consumption for 45 or 90 days.

Figure 1 displays the typical UV spectra of AH obtained from all the groups. The typical figures were characterized by variations in the UV range between 240 nm and 320 nm, where the intensity of the broadened peak was greater in all the food additive-consuming groups than in the control group. In addition, due to the administration of the preservative mixture (Fig. 1d), there was a significant change in the absorption discernible around 220–240 nm.

### Second derivative of typical UV-spectra of AH

**Fig. 2:**
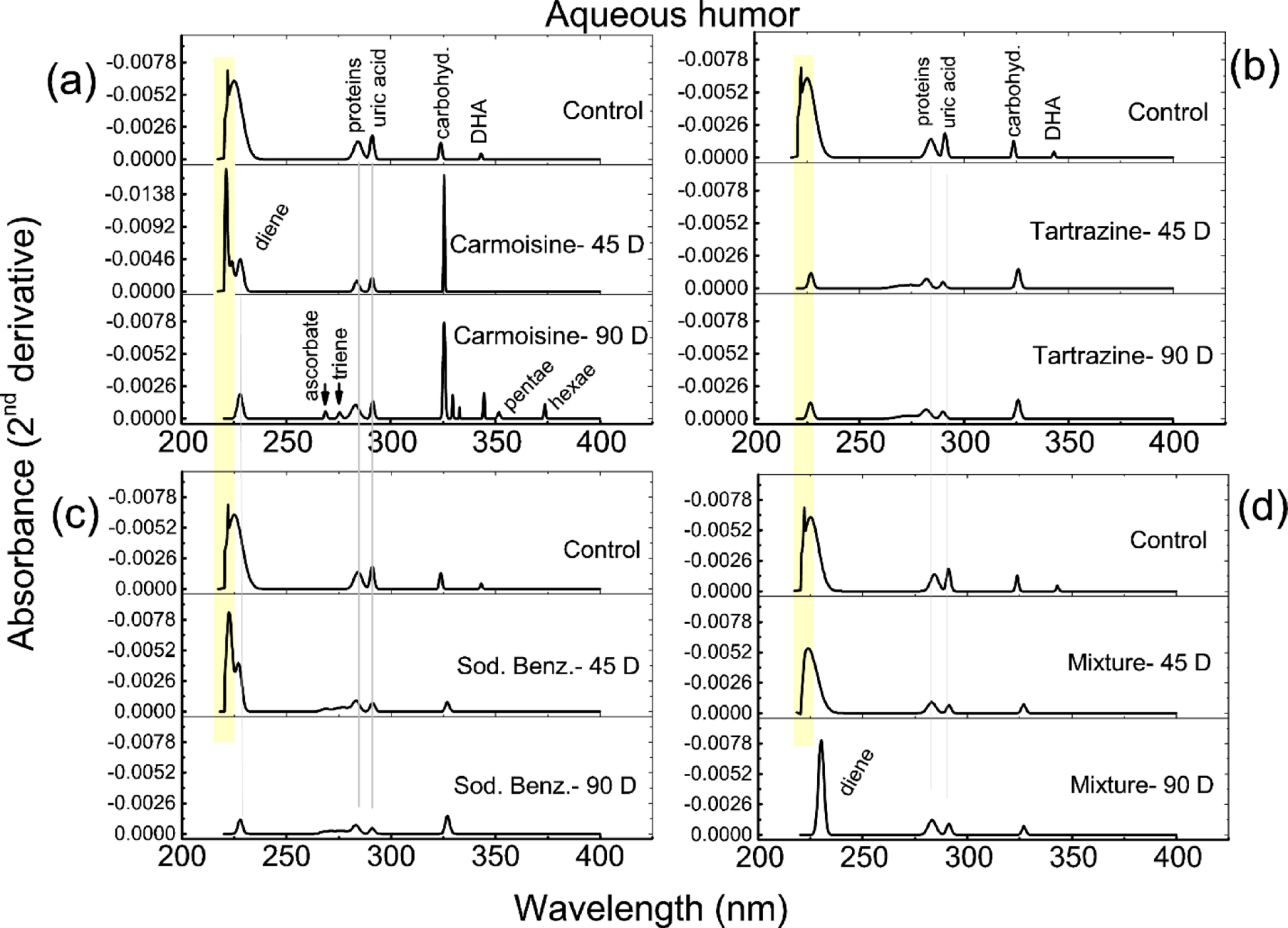
Calculated second derivative spectra of the aqueous humor of all the groups.

To resolve the typical absorption pattern of AH and detect its structural changes, the second derivative was calculated, revealing more distinct peaks, as displayed in Fig. 2. Generally, peptides and carboxylic acid moieties are discernible within the range of 220 nm to 225 nm, whereas the peak at approximately 285 nm corresponds to the aromatic ring portion of the proteins. Furthermore, the peak observed at 291 nm is attributed to uric acid, and absorption related to carbohydrates is evident at approximately 320 nm.

Comparing Figs. 1 and 2, it is clear that the second derivative UV spectroscopy method enhances spectral resolution and provides improved analytical capabilities over traditional UV spectroscopy. By reducing baseline noise and resolving overlapping peaks, it offers a more reliable approach to molecular analysis.

#### Administration of the colorant carmoisine

The control pattern was resolved into 6 peaks. Two adjacent peaks covered the 222–225 nm range, followed by two peaks located at 284 nm and 291 nm, while the higher wavelength peaks were found at 324 nm (carbohydrates) and 343 nm (deydroascorbic acid, DHA). The UV-spectral pattern resulting from daily consumption of carmoisine for 45 days resulted in a significant increase in absorption intensity (*p* < 0.001) for both peaks found at 222 nm (with an area percentage of 39) and 325 nm (with an area percentage of 14) compared with their control pattern counterparts, which were characterized by area percentages of 1.8 and 3.3, respectively (see Fig. 2a). The area percentages of the other two peaks related to proteins and uric acid decreased from 10 to 7 in the control pattern to 6 and 5, respectively. Additionally, one extra peak associated with diene conjugation was observed at 230 nm. Notably, the DHA peak was not observed.

Extending the administration period to 90 days resulted in significant changes, characterized by increased fragmentation with the observation of 11 peaks. A diene-conjugate peak was detected, along with the absence of absorption peaks at 222–225 nm. Furthermore, additional peaks emerged at 269 (ascorbate), 276 (triene conjugate), 330, 333, and 352 nm (pentaenoic conjugate). Compared with those of the control, the area percentages of the protein, uric acid, and carbohydrate peaks significantly increased (*p* < 0.001) to 17, 9, and 33, respectively. Additionally, another peak was resolved at 374 nm and attributed to the hexanenoic conjugate.

#### Administration of the colorant tartrazine

The second derivative of AH, as depicted in Fig. 2b, shows a diminished absorption intensity compared with that of the control pattern. Following daily consumption of tartrazine for either 45 days or 90 days, several consistent observations emerged: a reduction in the number of resolved peaks (4 peaks), a notable decrease (*p* < 0.001) in the calculated area percentage of the peaks discernible at 225 nm (22 and 23 after 45 and 90 days), a consistent protein peak (8 in both administered groups), and a uric acid peak (4 and 5 after 45 days or 90 days). Conversely, the carbohydrate peak area percentage displayed a different trend, significantly increasing (*p* < 0.001) to 27 and 24 after 45 and 90 days of daily consumption, respectively.

#### Administration of the sodium benzoate preservative

The interesting observation from Fig. 2c is the time-dependent decrease in the number of resolved peaks, from 5 to 4, with increasing daily consumption from 45 days to 90 days, respectively. In the absorption pattern of the 45-day group, the two peaks associated with peptides and carboxylic acid moieties exhibit a paradoxical response in their calculated area percentages: the first peak significantly increases (*p* < 0.001) to 50, whereas the second peak significantly decreases (*p* < 0.001) to 28 compared with their control counterparts. There was a consistent reduction (*p* < 0.01) in the area percentages of both proteins and uric acids to 6 and 4, respectively, with no change observed in the area percentage of the carbohydrate peak.

Extending the daily consumption to 90 days led to the detection of a diene conjugate peak at 230 nm, alongside the absence of absorption peaks in the 222–225 nm range. Furthermore, the area percentages of both the protein and uric acid peaks decreased to 5 and 3, respectively, with a significance level of *p* < 0.05. The percentage of carbohydrate peak area significantly increased to 27% compared with 3% in the control pattern (*p* < 0.001).

#### Administration of the food additive mixture

The effects of the co-consumption of both colorants (carmoisine and tartrazine) and the food preservative sodium benzoate on AH characteristics are depicted in Fig. 2d. Although four peaks can be resolved over the administration period, they exhibit distinct characteristics. After 45 days, the absorption intensity decreased relative to that of the control. Furthermore, a single broad peak is observed in the 222–225 nm range with λ_max_ = 225 nm, indicating a significantly increased area percentage (85, *p* < 0.001). Both peaks related to proteins and uric acid presented reduced area percentages (8.2 and 3.4, respectively), with a significance level less than 0.05. The area percentage of the carbohydrate peak matches that of its control counterpart.

With the extension of the mixture consumption period to 90 days, a diene peak was detected at 230 nm, accompanied by an increased area percentage of proteins (17 compared with 10 in the control pattern). There was a decrease in the area percentage of uric acid, which was found to be 4, compared with that of the control samples.

### Statistical evaluation

**Fig. 3:**
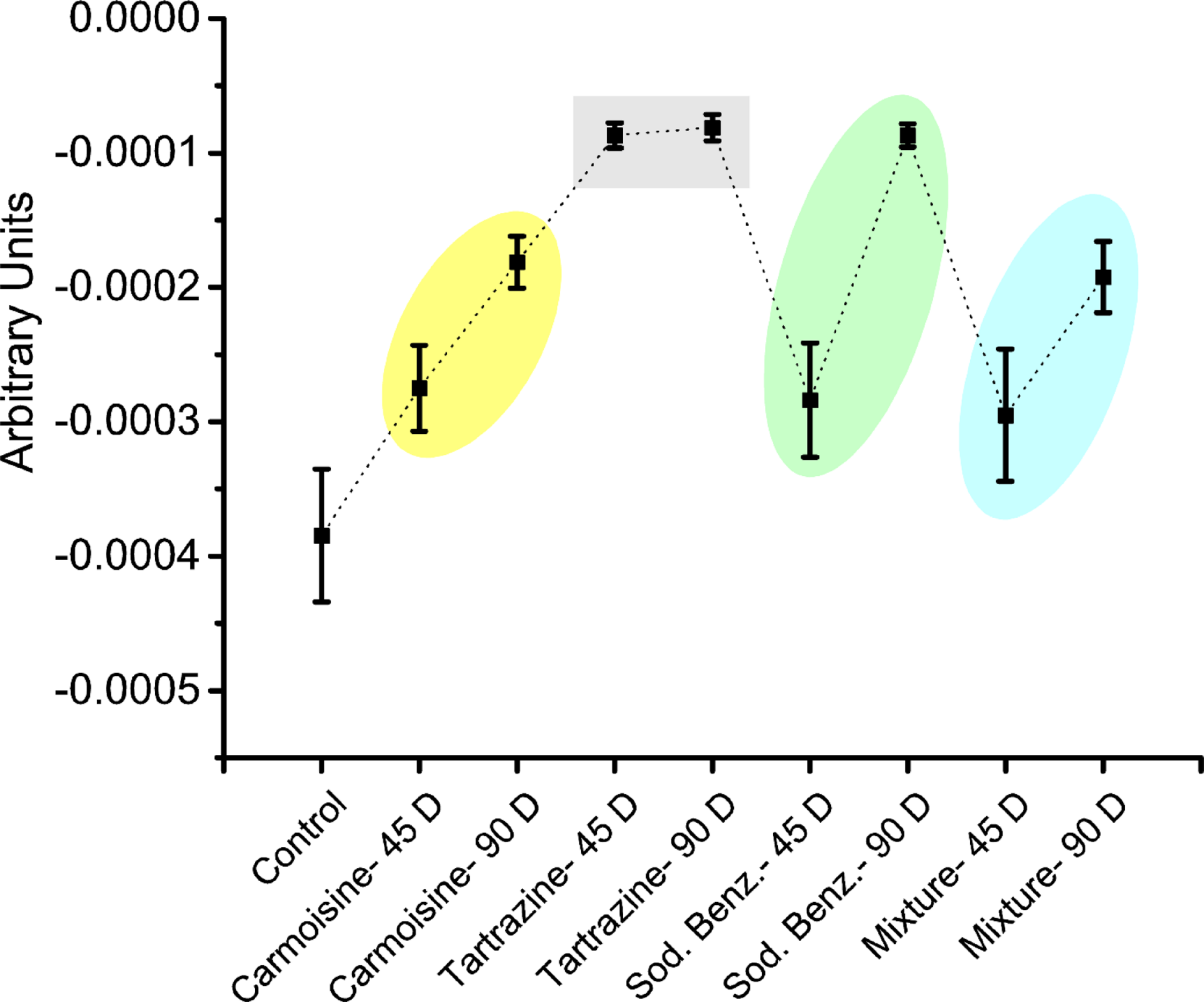
ANOVA-mean plots for second derivative spectra related to all studied groups.

Figure 3 depicts an ANOVA-means plot showing group means and error bars, visually highlighting differences in means due to food additive consumption over varying periods compared with the control. The second derivative spectra are time dependent, with the exception of the tartrazine groups.

**Fig. 4:**
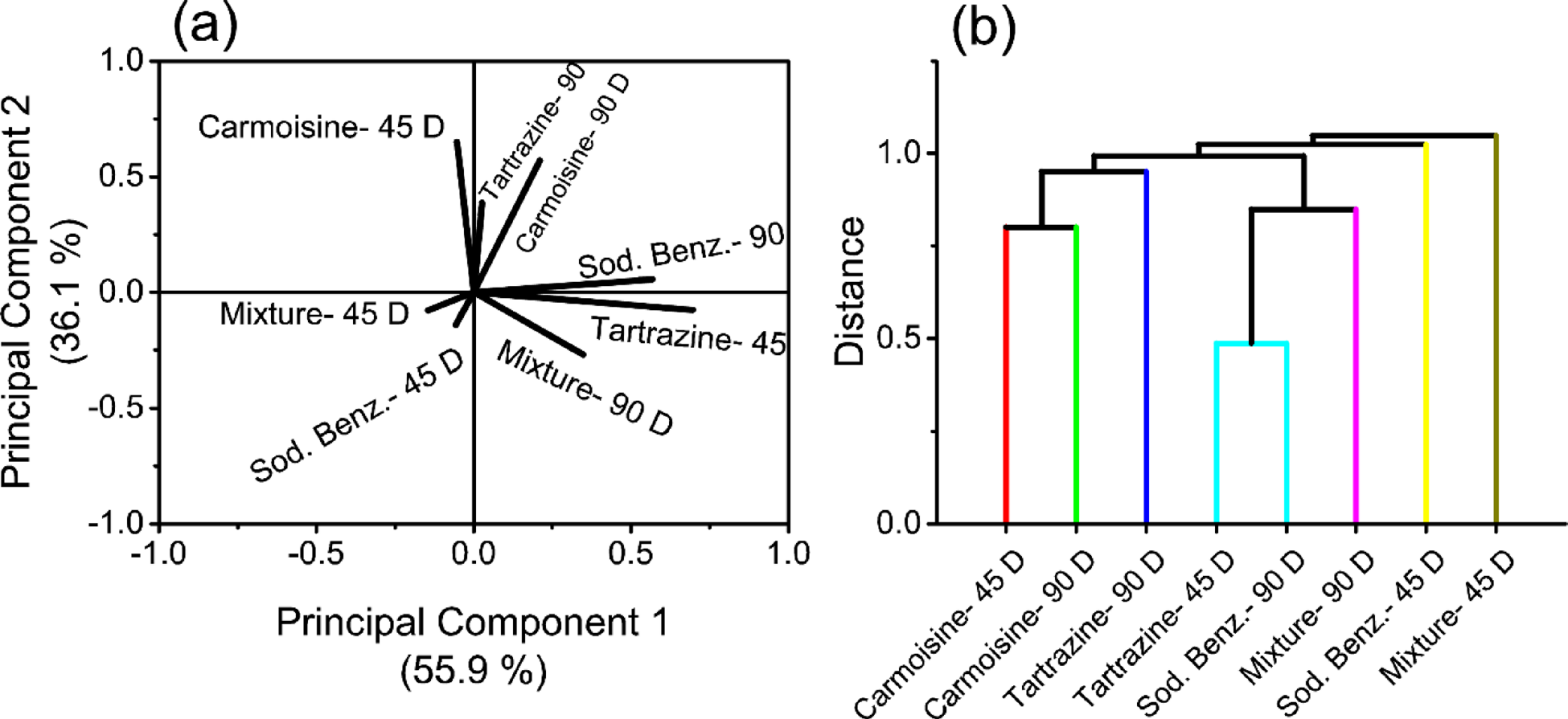
Chemometric analysis of aqueous humor spectra. (**a**) Loading plot of principal component analysis and (**b**) hierarchical cluster analysis.

Principal component analysis (PCA) is a method utilized to accentuate variation and uncover prominent patterns within a dataset, often facilitating easier exploration and visualization of the data. In Fig. 4a, the loading plot illustrates results where each group is represented by a line (vector). PC1 accounts for 55.9% of the variance, whereas PC2 accounts for 36.1%, collectively covering 92% of the data under analysis. Among the treated groups, PC1 positively influences the AH composition of Tartrazine-90 D, Carmoisine-90 D, Sodium benzoate-90 D, Tartrazine-45 D, and Mixture-90 D but negatively affects Carmoisine-45 D, Mixture-45 D, and Sodium benzoate-45 D. Conversely, PC2 positively affects the AH of Carmoisine-45 D, Tartrazine-90 D, Carmoisine-90 D, and sodium benzoate- 90 D but negatively impacts Mixture-45 D, Sodium benzoate-45 D, Tartrazine-45 D, and Mixture-90 D. Additionally, the analysis can be interpreted in terms of the cosine of the angle between vectors and their lengths. Notably, acute angles are observed between vectors representing the AH of carmoisine-consuming groups, whereas obtuse angles characterize those related to both sodium benzoate-consuming groups and mixture-consuming groups. Right angles are noted between the tartrazine-consuming groups. In terms of vector length, the shortest vectors are associated with Mixture-45 D, Sodium benzoate-45 D, and Tartrazine-90 D compared with their respective groups.

Hierarchical cluster analysis categorizes the variables into similar clusters, thus revealing the dissimilarity between groups, and results in a tree-diagram, i.e., a dendrogram, as depicted in Fig. 4b. The analysis revealed significant disparities in spectral changes among the investigated AH composition groups, which was consistent with the PCA findings. Notably, the groups administered carmoisine clustered together, whereas the other treated groups formed distinct clusters. Interestingly, the tartrazine-45D and sodium benzoate-90D groups shared a common cluster.

The results of the hierarchical cluster analysis provide evidence that the most significant structural variations observed in the AH of all investigated groups were associated with aromatic amino acids in proteins, specifically phenylalanine and tryptophan, within the absorption range of 260–289 nm. Additionally, when comparing groups receiving the same food preservative, the key observation in the carmoisine-treated groups was the presence of a pentanoic conjugate (330–352 nm). For the tartrazine and sodium benzoate groups, the variations were linked to the aromatic amino acids of proteins, with an additional carbohydrate peak at 337 nm observed exclusively in the sodium benzoate group. Lastly, the representative structural change in groups treated with a mixture of food additives was a diene conjugate peak at 230 nm.

## Discussion

The aqueous humor is a clear, watery fluid that fills the anterior chamber of the eye and is located between the cornea and the lens. It is essential for maintaining the shape of the eye and providing nutrients to avascular structures of the eye, such as the lens and cornea; therefore, the composition of the aqueous humor is crucial for maintaining proper vision and ocular health.

UV spectroscopy offers several advantages for diagnostic purposes. First, it allows for the discrimination of cancerous tissues from normal samples, providing distinctive resonance signatures of major biochemical components without fluorescence interference^22^. Additionally, UV-Vis spectroscopy can be utilized to study serum samples for disease diagnosis, aiding in understanding the biological nature of diseases such as diabetes^23^. UV spectroscopy can indeed be applied for the diagnosis of eye diseases^24,25^. The regenerative activity of conjunctival tumors can be rapidly assessed via spectrophotometry, aiding in preoperative or intraoperative diagnostics to determine the extent of surgical intervention needed and optimize treatment tactics^26^. Overall, its noninvasive nature, ability to provide clear spectral discrimination, and versatility make UV spectroscopy a valuable tool for the diagnosis of eye diseases.

The differentiated spectra illustrated in Fig. 2 reveal the existence of organic compounds, such as amino acids, proteins (240–310 nm) and carbohydrates (289–423 nm), alongside other chromophores that exhibit absorption within the same range as ascorbate, lipids and their derivatives. In the spectra of the control pattern resulting from the calculated secondary derivatives, a weak absorption peak emerged at approximately 343 nm, which was correlated with dehydroascorbic acid^27^. However, this peak was notably absent in the patterns depicted in Fig. 2(a-d), except for the Carmoisine-90 D group days. Dehydroascorbic acid (DHA), the oxidized form of vitamin C, can undergo reduction back to ascorbic acid through interactions with glutathione and NADH/NADPH, thereby contributing to its antioxidant activity^28,29^. Consequently, it serves as a reliable biomarker for oxidative stress in AH. The consumption of food additives for 45–90 days seems to diminish the antioxidant defense mechanisms of AH, potentially impacting both the ocular lens and cornea due to the potential for light-induced damage. Dehydroascorbic acid is transported into the aqueous humor via a Na+-independent mechanism and is subsequently converted intracellularly back to ascorbic acid by DHA reductase, offering protection against light-induced damage^30^. The decreased or absent DHA peak suggests prior redox imbalance and insufficient recycling capacity^31–33^. Previous studies have shown that high DHA levels can contribute to oxidative stress, as DHA is a less effective antioxidant than ascorbic acid^34^. An imbalance between DHA and ascorbic acid may lead to increased reactive oxygen species formation, potentially damaging corneal epithelial cells and other ocular tissues^35^.

In another and related context to the antioxidant activity within AH, uric acid in addition to its significance role in various eye conditions, can serve as a biomarker for ocular conditions and could be targeted for therapeutic interventions in diseases such as posterior subcapsular cataract (PSC) and diabetic macular edema (DME). Elevated levels of uric acid in the aqueous humor are associated with the pathogenesis of PSC^36^. Additionally, uric acid levels in the aqueous humor have been linked to DME, with higher levels correlating with more severe DME cases^37^. Furthermore, uric acid has been identified as a potent endogenous antioxidant in the eye that potentially influences the development of cataracts^38^. The results displayed in Fig. 2 regarding the uric acid peak (291 nm) are consistent with the previous results of dehydroascorbic acid, which indicates that daily consumption of different food additives diminishes the antioxidant activity of AH by reducing the area percentage of its corresponding peaks in most studied groups. The conflicting findings within the Carmoisine-90 D group, where an increase in the area percentage of both the DHA and uric acid peaks was observed, suggest that this colorant additive may exert its unique impact based on the duration of consumption. Moreover, the increased antioxidant activity of AH could be associated with the induction of moderate to severe oxidative stress.

The induced oxidative stress is clearly observed as a result of carmoisine consumption, which is dependent on duration. The newly detected peak of the conjugated dienoic system (–C =C–C=C–, 230 nm) reflects a higher content of molecules with two conjugated double bonds. After 90 days, the unsaturated molecules extend to include other molecules with trienoic (276 nm), pentaenoic (352 nm), and hexaenoic (374 nm) conjugated systems. Conjugated dienoic, trienoic, pentaenoic, and hexaenoic systems in unsaturated molecules serve as indicators of oxidative stress^39,40^. These conjugated structures are formed as a result of oxidative attack on polyunsaturated fatty acids, leading to the generation of chromophoric triene and tetraene structures^41^, and their estimation provides an accurate assessment of oxidative stress levels both in vitro and in vivo^42^. This result, together with the detection of the ascorbate peak (269 nm), implies that the eye is under toxification from different free radicals. The consumption of tartrazine does not impact the unsaturation profile of AH. However, daily consumption of sodium benzoate or a blend of food additives over 90 days triggers oxidative stress, particularly noticeable in the Mixture-90 D group (diene peak at λ_max_ = 230 nm), which may be linked to Carmoisine.

The findings depicted in Fig. 2 indicate that the consumption of food additives, either individually or in combination, elicits distinct effects on the structure and concentration of proteins in aqueous humor fluid. Specifically, the impact of the colorant tartrazine aligns consistently with the duration of consumption, as evidenced by the similar characteristics of both peaks (225 nm and 284 nm) in terms of area percentage. In contrast, the colorant carmoisine exhibits effects on proteins that do not correlate with the consumption period, a pattern also observed with sodium benzoate consumption. Moreover, the protein alterations observed in the Mixture-45 D group can be attributed to both carmoisine and sodium benzoate consumption, whereas those in the Mixture-90 D group are linked to sodium benzoate intake.

Carbohydrates play a significant biological role in the aqueous humor. Studies have shown that the aqueous humor contains various protein-bound carbohydrates, such as hexose, hexosamine, sialic acid, glucose, fructose, and glycogen^43–45^. These carbohydrates are essential for maintaining the transparency and structure of the cornea and act as nutrient metabolites^46^. Furthermore, in conditions such as wet age-related macular degeneration and diabetic cataracts, alterations in glucose metabolism and carbohydrate levels in the aqueous humor have been observed, indicating potential pathological mechanisms related to glucose metabolism^47^ and shifts in energy and amino acid metabolic pathways. Therefore, the detected carbohydrate peak (approximately 323 nm) in AH highlights its crucial role in ocular health and disease pathogenesis and can also be used as a diagnostic probe. Both colorant additives, carmoisine and tartrazine, significantly increased the absorption intensity of the carbohydrate peak, and accordingly, the area percentage with carmoisine groups displayed the higher area percentage. Therefore, the metabolic changes associated with colorant additives are more common during carmoisine consumption than during tartrazine consumption, regardless of the consumption period. Daily consumption of sodium benzoate for 90 days was found to affect the metabolic state of the eye.

The loading plot displayed in Fig. 4a provides an additional perspective on the obtained results. The length of the vectors serves as a measure of the administration period’s discriminating ability on the composition of AH, indicating the magnitude of interaction. The similarity in length between the vectors associated with carmoisine-consuming groups suggests a consistent interaction magnitude irrespective of the consumption period. In contrast, the Tartrazine-45 D group presented a longer vector than did the Tartrazine-90 D group, indicating more pronounced effects after 45 days than after 90 days of consumption. This suggests that changes in AH composition initiated within the first 45 days of consumption persist for up to 90 days without further amplification in interaction magnitude.

Compared with the consumption of tartrazine, the daily consumption of the preservative sodium benzoate has contrasting effects, with the magnitude of the interaction being dependent on time. This observation extends to groups consuming mixtures of food additives, indicating that when sodium benzoate is part of the mixture, its effects outweigh those of co-added preservatives.

Furthermore, the angle between the vectors determines the direction of interaction. This direction of interaction is evident in the dendrogram in Fig. 4b, where the interactions between AH and carmoisine exhibit similar directions, forming one cluster. The remaining investigated additives form individual clusters, reflecting unique effects or additive characteristics. Notably, the observed changes in Tartrazine-45 D resemble those induced by sodium benzoate-90 D.

Now, and at this point, the pressing question that needs addressing is as follows: What truly defines the essence of these alterations in AH composition? It is widely acknowledged that aqueous humor experiences consistent turnover rates, estimated at 0.1 µl/min in rats^48^, 3.9 µl/min in rabbits^49^, and 2.4 ± 0.6 µl /min in humans, typically approximately 3.0 µl/min in the morning, 2.4 µl/min in the afternoon, and decreasing to 1.5 µl/min at night^50^. This suggests that the composition of AH undergoes daily alterations, rendering the changes observed in this study transient, unlikely to persist for 45 days, or even extend to 90 days of food additive consumption. Hence, the aforementioned variations do not directly result from the ingestion of food additives but rather from their influence on the formation and composition of AH. Aqueous humor dynamics, encompassing production and outflow, are intricately linked to the ciliary body -the site of formation- and the trabecular meshwork and the uveoscleral pathway, the primary locations of aqueous humor outflow. The formation process involves three mechanisms: diffusion, ultrafiltration, and active secretion^51–53^. These mechanisms are collectively or individually influenced by the daily consumption of food additives. The current results highlight the role of the diffusion mechanism in aqueous humor dynamics, in contrast to ultrafiltration, which plays a lesser role compared to active secretion in determining AH volume. Active secretion remains the primary process by which sodium and other solutes cross the blood-aqueous barrier into the AH^54–56^. Within the diffusion-driven component of AH secretion, certain metabolites and ions passively diffuse from the plasma into the AH. The presence of metabolites such as ascorbate in AH suggests the involvement of selective transport mechanisms. Furthermore, increased oxidative stress can compromise the integrity of the blood-aqueous barrier by damaging endothelial cells in the ciliary body and trabecular meshwork, thereby contributing to alterations in AH composition^57,58^.

Aqueous humor dynamics differ significantly between rats and humans. In rats, a conventional outflow facility of approximately 0.023 µL/min/mm Hg, representing an intermediate level compared to humans. In contrast, human aqueous humor dynamics are more complex, involving both conventional and unconventional outflow pathways, with these mechanisms undergoing distinct changes with aging^48,59,60^.

## Conclusions

UV-second derivative spectroscopy has emerged as a potent investigative tool, providing crucial insights into the health status of the eye. It reveals that the effects observed in the constituents of aqueous humor due to daily consumption of allowable doses of food additives are directly connected to the ciliary body, the site of AH formation. The effects attributed to carmoisine ingestion are time dependent, particularly concerning lipid–unsaturated conjugates. In contrast, those linked to the other colorant dye, tartrazine, do not vary with the time of consumption. For the preservative sodium benzoate, the observed changes are contingent upon the consumption period. Additionally, the eye is under toxicity due to the diminished antioxidant activity of both DHA and uric acid. Chemometric analysis has further deepened the understanding of these observed changes and their relationship with the period of consumption.

## Electronic supplementary material

Below is the link to the electronic supplementary material.

Supplementary Material 1.

## Data Availability

All data generated or analyzed during this study are included in this published article, and its supplementary information files.
